# Close interactions between lncRNAs, lipid metabolism and ferroptosis in cancer

**DOI:** 10.7150/ijbs.66181

**Published:** 2021-10-25

**Authors:** Jingjing Huang, Jin Wang, Hua He, Zichen Huang, Sufang Wu, Chao Chen, Wenbing Liu, Li Xie, Yongguang Tao, Li Cong, Yiqun Jiang

**Affiliations:** 1 The Key Laboratory of Model Animal and Stem Cell Biology in Hunan Province, Hunan Normal University, Changsha, 410013 Hunan, China.; 2 School of Medicine, Hunan Normal University, Changsha, 410013 Hunan, China.; 3 School of Medicine & Holistic Integrative Medicine, Nanjing University of Chinese Medicine, Nanjing, 210013 Jiangsu, China.; 4 Department of Head and Neck Surgery, The Affiliated Cancer Hospital of Xiangya School of Medicine, Central South University, Changsha, 410013 Hunan, P.R. China.; 5 Key Laboratory of Carcinogenesis and Cancer Invasion, Ministry of Education, Department of Pathology, Xiangya Hospital, School of Basic Medicine, Central South University, Changsha, 410078 Hunan, China.

**Keywords:** LncRNAs, Lipid metabolism, Ferroptosis, Cancer

## Abstract

Abnormal lipid metabolism including synthesis, uptake, modification, degradation and transport has been considered a hallmark of malignant tumors and contributes to the supply of substances and energy for rapid cell growth. Meanwhile, abnormal lipid metabolism is also associated with lipid peroxidation, which plays an important role in a newly discovered type of regulated cell death termed ferroptosis. Long noncoding RNAs (lncRNAs) have been proven to be associated with the occurrence and progression of cancer. Growing evidence indicates that lncRNAs are key regulators of abnormal lipid metabolism and ferroptosis in cancer. In this review, we mainly summarized the mechanism by which lncRNAs regulate aberrant lipid metabolism in cancer, illustrated that lipid metabolism can also influence the expression of lncRNAs, and discussed the mechanism by which lncRNAs affect ferroptosis. A comprehensive understanding of the interactions between lncRNAs, lipid metabolism and ferroptosis could help us to develop novel strategies for precise cancer treatment in the future.

## Introduction

During carcinogenesis, cancer cells usually exhibit a series of metabolic abnormalities that support their need for growth and metastasis. In recent years, increasing evidence has shown that lipid metabolism can be reprogrammed in cancer cells 1, 2, which greatly affects the proliferation, invasion and migration of cancer cells. Lipids are hydrophobic molecules that contain thousands of different types of molecules, including cholesterol, fatty acids (FAs), triacylglycerol (TG) and phospholipids (PL) 3, 4. These compounds play crucial roles in various biological processes, such as the biosynthesis of membrane lipids, energy metabolism, storage and signal transduction 4, 5. Cancer cells cause dysregulation of lipid metabolism by affecting the synthesis, uptake, modification, degradation, and transport of these lipids in cells, thus affecting their normal physiological function 3, 6. In addition, a growing body of studies has found that aberrant lipid metabolism is closely related to ferroptosis, a new type of cell death.

Ferroptosis is a novel form of programmed cell death caused by lipid peroxidation (LPO) 7, which is characterized by the accumulation of LPO products induced by lethal reactive oxygen species (ROS) 8. Polyunsaturated free fatty acids (PUFAs) are rich in cell membranes and organelle membranes and tend to react with ROS and cause cell damage 9. Since saturated membrane lipids are not sensitive to peroxidation, highly saturated membrane lipids help protect tumor cells from ROS damage. In tumors, tumor cells can express high levels of antioxidant proteins to reduce ROS levels and prevent the destruction of redox homeostasis 10. Therefore, the discovery of ferroptosis may provide more ways to treat cancer.

Long noncoding RNAs (lncRNAs) are broadly defined as transcripts of more than 200 nucleotides that are not translated into proteins; lncRNAs were historically considered junk DNA and not taken seriously. However, in recent years, it has been increasingly found that lncRNAs have a place in the occurrence and development of cancer 11. LncRNAs have complex secondary and tertiary structures and diverse subcellular localizations. On the basis of their subcellular localization, lncRNAs can be classified as nuclear, cytoplasmic, and mitochondrial lncRNAs 12. Functionally, nuclear lncRNAs seem to preferentially play roles in chromatin remodeling, transcriptional regulation and RNA processing, while cytoplasmic lncRNAs can modulate the stability or translation of mRNA and influence the cell signal cascade 13. Mitochondrial lncRNAs may act as retrograde signaling molecules, coordinating gene expression in the nucleus and mitochondria and ultimately triggering cellular signaling pathways 14. Many lncRNAs have been regarded as crucial factors in cancer development by affecting a series of cellular processes, including cell proliferation 15, 16, differentiation 17, 18, metastasis 19, 20 and apoptosis 21, 22, at different levels. Therefore, as a regulatory factor, lncRNAs can regulate tumor lipid metabolism and ferroptosis in carcinoma by mediating the expression of enzymes related to lipid metabolism and ferroptosis-related genes.

Therefore, this article will discuss three major points: 1) several abnormal lipid metabolisms (cholesterol, FAs, TG and PL) regulated by lncRNAs; 2) the effect of lipid metabolism on the expression of lncRNAs; and 3) lncRNAs affecting ferroptosis through lipid metabolism. Elaborating the regulatory relationships of these three factors provides new therapeutic targets and ideas for cancer treatment, which may help alleviate the current clinical difficulties in the treatment of cancer.

## LncRNAs associated with cholesterol reprogramming in cancer

**Figure 1:**
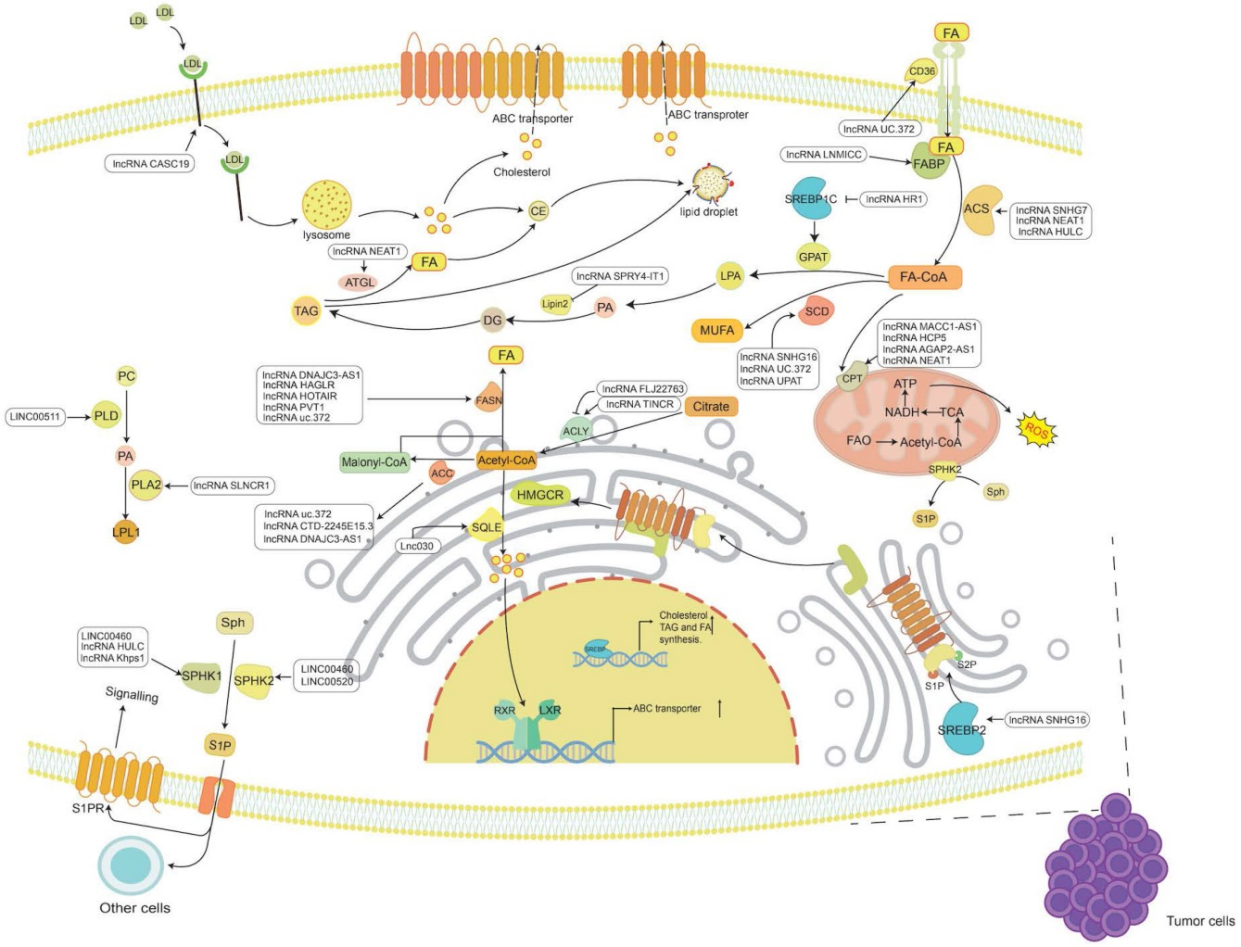
** The known functions of lncRNAs in tumor lipid metabolism.** The main lipids in tumor lipid metabolism include cholesterol, FAs, TG and PL. LncRNAs regulate the synthesis and catabolism of these four types of lipids through a variety of mechanisms, thereby participating in the occurrence and development of cancer.

Cholesterol metabolism is vital for cellular and systemic biological functions. For instance, cholesterol regulates membrane fluidity and permeability, is an essential component in mammalian cell membranes, and is the precursor of bile acid, cholecalciferol and steroid hormones 23. Dysregulated cholesterol balance not only promotes cardiovascular disease but also increases the risk of occurrence of other diseases, such as neurodegenerative diseases and cancers 24. Normally, cellular cholesterol metabolism reflects the dynamic balance between synthesis, uptake, storage and efflux 3. Over the past decade, the cancer research community has witnessed growing interest in cholesterol reprogramming, including the study of how cancer cells reprogram their cholesterol metabolism, how these reprogramming-derived metabolites consequently promote the progression of cancer and the identities of the key regulators in cholesterol reprogramming 25, 26. LncRNAs can interact with RNA, chromatin and protein, thus modulating mRNA stability, chromatin structure and the function of proteins (including transcription factors), which makes lncRNAs an important group of crucial factors governing cholesterol metabolism processes in cancer 27 (Figure 1 and Table 1).

### LncRNAs regulate cholesterol synthesize and uptake

Cholesterol is an indispensable substance in the human body and can be synthesized *de novo* or ingested from an external source 28. Acetyl-CoA and NADPH are the basic raw materials for cholesterol synthesis. Acetyl-CoA can be reduced to mevalonate (MVA) under a series of enzymatic reactions with hydroxymethylglutaryl-coenzyme A reductase (HMGCR) as the key enzyme. Then, MVA undergoes decarboxylation and phosphorylation to generate isoprenoids 29. After the condensation of isoprenoids, 30-carbon squalene is formed. Then, squalene is cyclized to lanosterol under the action of squalene epoxidase (SQLE) 30. Finally, lanosterol undergoes oxidation, decarboxylation and reduction reactions to generate cholesterol 31. When the synthesis of cholesterol is insufficient, the low-density lipoprotein receptor (LDLR) can mediate exogenous absorption to maintain the balance of cholesterol in the body 32. In addition to the increase in cholesterol, which can provide growth requirements for tumor cells, the isoprenoid produced in the MVA pathway can also contribute to the occurrence and development of cancer 33. Isoprenoids can posttranslationally prenylate small GTP-binding proteins (GTPases), such as the Ras and Rho families, and give them carcinogenic capacity through specific binding to the cell membrane 34. It is worth mentioning that isopentenyl diphosphate (IPP), the isoprenoid precursor produced in the MCV pathway, can mediate the synthesis of glutathione peroxidase 4 (GPX4) and participate in cholesterol oxidation 35-37. The hydrogen atom at position C-7 in cholesterol can be taken up by free radicals and then react with diatomic oxygen to produce cholesterol peroxide, leading to lipid peroxidation. However, GPX4 can reduce the occurrence of lipid peroxidation by converting lipid peroxides into lipid alcohols under its antioxidant effect 38, 39. Therefore, studying the mechanism of the effect of lncRNA on the synthesis and uptake of cholesterol can find ways to inhibit cholesterol reprogramming so that it cannot meet the growth conditions of tumor cells.

It has been reported that lncRNAs can control the expression of HMGCR and LDLR by regulating sterol regulatory element-binding protein 2 (SREBP2) 40. SREBPs are a class of transcription factors controlling lipid homeostasis by modulating the expression of enzymes required for lipogenesis. SREBP includes three subtypes: SREBP-1a, SREBP-1c, and SREBP-2. It has been indicated that SREBP2 seems preferential to activate the transcription of genes involved in cholesterol synthesis 41-43. When cholesterol is insufficient, endoplasmic reticulum (ER) cholesterol, as a sensor of intracellular cholesterol homeostasis, can trigger the transport of SREBP2 from the ER to the Golgi apparatus and then to the nucleus to directly regulate the expression of cholesterol-related HMGCR and LDLR 23, 40. Therefore, directly inhibiting SREBP2 through cholesterol starvation may be an effective strategy against cancer 44. Yu et al. found that *lncRNA SNHG16* is highly expressed in pancreatic cancer and can directly sponge *miR-195* to regulate the expression of *SREBP2* to accelerate the progression of pancreatic cancer. Moreover, the reduction in pancreatic cancer cell adipogenesis after inhibiting the expression of *SREBP2* further proves that *lncRNA SNHG16* may fuel the growth of pancreatic cancer cells by regulating the *miR-195/SREBP2* axis to provide an energy supply 45.

SQLE is another key controlling enzyme in the MVA pathway. It has been confirmed that *SQLE* is a metabolic oncogene in breast cancer and is associated with a poor prognosis of breast cancer (BC) 46. Recent studies have discovered that *lnc030* is highly expressed in breast cancer stem cells (BCSCs) and can work with poly(rC)-binding protein 2 (PCBP2) to stabilize the expression of *SQLE*. PCBP2 is an RNA-binding protein that acts as an intermediary between lnc030 and SQLE. PCBP2 promotes the stability of *SQLE* mRNA and the production of cholesterol by combining with the *lnc030* fragment 301-412 nt and the 3'-UTR of *SQLE* to form a ternary complex. It has been reported that increased cholesterol synthesis activates PI3K/AKT, the consistent cancer signaling pathway, which is involved in BCSC stemness maintenance 47. Therefore, *lnc030* can promote the occurrence and growth of BC through the SQLE/cholesterol/PI3K/AKT signaling axis.

In addition, lncRNAs can also directly affect the expression of LDLR to promote tumor development. Wang et al. found that *lncRNA CASC19* was significantly upregulated in non-small cell lung cancer (NSCLC) and was positively correlated with the proliferation and metastasis of NSCLC cells. It was predicted and proved that *LDLR* is the downstream target gene of *miR-301b-3p* in NSCLC, and it is inhibited when interacting with *miR-301b-3p*. However, *CASC19* can sponge *miR-301b-3p* and restore the effect of LDLR. Therefore, *CASC19* plays a role in NSCLC by targeting the *miR-301b-3p/LDLR* axis to promote the proliferation and metastasis of cancer cells 48.

### LncRNAs regulate cholesterol efflux

Most peripheral cells and tissues lack the ability to metabolize cholesterol 49. When the cholesterol in the cell exceeds what the cell needs, cholesterol is either converted to cholesterol ester (CE) by the action of cholesterol acyltransferase (ACAT) 50, which is stored in lipid droplets or secreted in lipoproteins, or is excreted from the cell via an ATP binding cassette (ABC) transporter. Liver X receptors (LXRs) can be used as cholesterol sensors 51. When intracellular cholesterol is at a high level, LXRs are activated by cholesterol derivative oxysterols 52, and then they can bind to retinoid X receptor alpha (RXRα) as an obligate heterodimerization partner to direct repeat 4 (DR4) in the promoter of ABC transporters 53, 54, thereby activating the expression of ABC transporters to promote cholesterol export.

LXR is composed of alpha and beta subtypes. A number of studies have shown that the activation of LXR can significantly inhibit tumor progression. For example, activating the expression of LXR can inhibit the protein associated with cell proliferation in BC 55. Activating LXR can block the G1 phase of cancer cells in intestinal tumors and increase caspase-dependent apoptosis to inhibit the development of cancer 56. In addition, LXR-623, as an activator of LXR, can effectively inhibit the development of glioblastoma (GBM) by activating LXRβ, lowering cholesterol levels, and inducing apoptosis 57. ATP binding box transporter A1 (ABCA1), a subtype of ABC, can be activated by LXRs to promote cholesterol efflux 58. When ABCA1 expression is reduced, cholesterol is allowed to accumulate in cells. Cholesterol accumulation leads to reduced membrane fluidity and inhibition of the mitochondrial permeability transition (MPT), which prevents the release of cell death-promoting molecules in mitochondria 59, ultimately promoting the progression of cancer. However, the current research on the regulatory mechanism of lncRNAs on ABC is mostly limited to noncancer diseases. For example, *lncRNA GAS5* is highly expressed in atherosclerosis and can recruit enhancer of zeste homolog 2 (EZH2) to the *ABCA1* promoter region to promote histone methylation modification of *ABCA1*. Eventually, transcription of *ABCA1* is inhibited, leading to cholesterol accumulation and atherosclerosis 60. In addition, *lncRNA ENST00000602558.1* can directly bind to P65 to promote P65 binding to the promoter of *ABCG1* and inhibit the expression of *ABCG1* mRNA and protein in vascular smooth muscle cells. The reduction in cholesterol efflux mediated by ABCG1 further leads to dyslipidemia and atherosclerosis 61.

## LncRNAs mediate abnormal fatty acid metabolism in cancer

Many lipids are synthesized from FAs, a class of molecules consisting of hydrocarbon chains of varying lengths and degrees of desaturation 6. Within cells, FAs have various functions, including being a component of the membrane, conducting signals, or being oxidized to release energy. FAs are either obtained from an external source or synthesized from scratch. Under normal conditions, most normal cells preferentially use exogenous FAs to meet their lipid requirements 62. FAs must be activated by covalent modification of fatty acyl-CoA synthetase (ACS) to synthesize lipids or decompose to produce energy 63. In cancer, FA metabolism is undoubtedly altered due to the high energy requirements of rapidly proliferating cells 6, 64, 65. Therefore, the roles of lncRNAs in FA uptake, *de novo* synthesis, and oxidative degradation in tumor progression are also worthy of study and discussion (Figure 1 and Table 1).

### LncRNAs regulate the *de novo* synthesis of fatty acids

Most normal cells have the ability to take up lipids from the extracellular environment and are thus more inclined to take up FA exogenously^62^, ^66^. In contrast, an increase in the demand for lipids by tumor cells has been observed in tumors, such as signaling molecules and membrane biosynthesis. Therefore, the *de novo* synthesis of FA plays a dominant role in tumors 67-69. The increased demand for FA synthesis becomes an adaptation to the high metabolic demand of cancer cells. The *de novo* synthesis of FAs requires the action of a variety of enzymes. First, glucose is decomposed into pyruvate and then enters the mitochondria to oxidize and decarboxylate to generate acetyl-CoA; since acetyl-CoA cannot directly penetrate the mitochondrial membrane and needs to synthesize FAs in the cytoplasm, it must first condense with oxaloacetate to form citric acid and then enter the cytoplasm. Then, acetyl-CoA is generated under the action of ATP citrate lyase (ACLY), and finally, saturated FAs are synthesized under the continuous condensation reaction of acetyl-CoA carboxylase (ACC) carboxylation and fatty acid synthase (FASN) 64, 70. Additionally, saturated FAs can be desaturated by stearoyl-CoA desaturase (SCD) according to cellular requirements to produce monounsaturated FAs 64. Therefore, inhibiting these enzymes and reducing FA synthesis will effectively limit the growth of cancer cells.

#### ATP citrate lyase

ATP citrate lyase (ACLY) is an enzyme that catalyzes an important step in FA synthesis. It can catalyze the conversion of citric acid and coenzyme A (CoA) to acetyl-CoA and oxaloacetic acid. Acetyl-CoA is essential for FA synthesis and cancer cell proliferation. ACLY is abnormally expressed and active in many tumors 71, 72, such as renal carcinoma 73, pancreatic cancer 74, BC 75 and gastric cancer (GC) 76.

Zheng et al. found that *lncRNA TINCR* was highly expressed in nasopharyngeal carcinoma (NPC) cells. RNA pull-down assay and RNA-EMSA analysis confirmed that the 1-876 nt region of *TINCR* can bind ACLY. Subsequently, it was shown that *TINCR* can inhibit the ubiquitin-mediated degradation of ACLY to protect the proteasome-dependent degradation of ACLY, thereby maintaining the stability of the ACLY protein. The increased stability of the ACLY protein increases the level of acetyl-CoA, hence increasing the content of free fatty acids (FFAs), leading to the increased proliferation, metastasis and chemoresistance of cancer cells 77. Zhang et al. demonstrated that *lncRNA FLJ22763* is downregulated in GC tissue and is closely related to the survival of patients. The researchers revealed a significant negative correlation between *FLJ22763* and *ACLY* mRNAs in GC. After *FLJ22763* overexpression, *ACLY* was significantly downregulated, hence inhibiting GC cell malignancy and xenograft tumor growth 78. In addition, in recent years, researchers have used the statistical method of weighted correlation network analysis (WGCNA) to determine the coexpression network of lncRNAs and ACLY in GC. WGCNA can identify the genes most related to cancer in the database. Then, a series of methods, including univariate and multivariate Cox regression analyses and Pearson correlation and hypergeometric tests, confirmed that ACLY was correlated with *lncRNA PPP1R26-AS1*, *lncRNA DLEU1* and *lncRNA TMPO-AS1* 79. However, further experiments are needed to verify the relationship between these lncRNAs and ACLY.

In addition, some lncRNAs that regulate the expression of ACLY are also associated with viral infections. Lipid production has a positive effect on virus replication 80. *Linc-Pint* is significantly downregulated in HCV-infected hepatocytes. *Linc-Pint* overexpression may downregulate the protein level of serine/arginine protein-specific kinase 2 (SRPK2) through proteasome degradation 81. SRPK2 activates serine/arginine (SR) proteins involved in mRNA splicing and maturation 82. It has been reported that SRPK2 can promote effective splicing by the phosphorylation of SR proteins to increase the stability of *ACLY* and *FASN* mRNA 83. Therefore, the overexpression of *Linc-Pint* decreased the expression of SRPK2, which further reduce the stability of *ACLY* and *FASN* mRNA. Finally, the HCV-induced lipid production pathway is inhibited to limit the replication of HCV 81.

#### Acetyl-CoA carboxylase

Acetyl-CoA can be carboxylated to malonyl-CoA under the action of acetyl-CoA carboxylase (ACC), which is an indispensable step in FA synthesis 3. ACC has two different subtypes, ACC1 and ACC2, and the functions of malonyl-CoA produced by them are different. The malonyl-CoA produced under the action of ACC1 mainly promotes the synthesis of FAs, while the malonyl-CoA produced under the action of ACC2 mainly inhibits the oxidation of FAs 84, keeping the FA content in the body at a high level. To further meet the needs of tumor cells, ACC is highly expressed in NSCLC 85, cervical squamous cell carcinoma 86 and colorectal cancer 87.

The mechanisms by which lncRNAs regulate ACC in different tumors are also different. *LncRNA DNAJC3-AS1* is highly expressed in colorectal cancer and can regulate the expression of ACC and FASN by activating PI3K/AKT, a recognized oncogenic pathway, thereby promoting tumor progression 87. Recent studies have demonstrated that *lncRNA CTD-2245E15.3* is highly expressed in NSCLC, and its inhibition can regulate lipid metabolism-related genes. Based on this conclusion, the study also showed that the knockout of *CTD-2245E15.3* can phosphorylate the Ser117 site of ACC1. The Ser117 site of ACC1 is the inhibitory site of enzyme activity. Its phosphorylation inhibits the enzyme activity of ACC1, which further leads to lipid synthesis obstacles and ultimately inhibits tumor growth. It is concluded that *CTD-2245E15.3* promotes the progression of non-small cell lung cancer by regulating the enzyme activity of ACC1 85.

#### Fatty acid synthase

Fatty acid synthase (FASN) is a multifunctional enzyme that catalyzes the biosynthesis of palmitate esters in an NADPH-dependent manner and thus participates in the synthesis of FAs 88. FASN is widely expressed in normal cells, and its promotion of expression leads to an increase in FA synthesis. An imbalance in FASN expression can cause many diseases. For example, the high expression of FASN has been demonstrated to be closely related to poor prognosis in various cancers, such as BC 89 and NSCLC 90.

*HAGLR* is a lncRNA transcribed from the *HOXD* cluster on human chromosome 2 that is upregulated in multiple cancers, including colon cancer 91, hepatocellular carcinoma 92 and BC 93, and is closely related to progression and unfavorable prognosis. Lu et al. discovered that the level of *HAGLR* expression in NSCLC increased and was associated with poor prognosis in patients. The level of FASN is positively correlated with the expression of *HAGLR*. The expression of FASN in NSCLC decreased with the knockdown of *HAGLR*, which reduced the FFA content in cells and inhibited the proliferation, invasion and tumorigenesis of NSCLC cells 94. In addition, *lncRNA HOTAIR* plays a role in regulating chromatin dynamics in gene regulation. As a proto-oncogene, it is highly expressed in a variety of cancers 95. Knockout of *HOTAIR* can reduce FASN expression and inhibit FA synthesis in NPC cells, thereby inhibiting their proliferation and invasion 96. It is worth mentioning that matrix metalloproteinase-9 (MMP-9), as a potential cancer marker 97, has been downregulated in the knockout of *HAGLR* and *HOTAIR* 94, 96. Therefore, whether FASN affects MMP-9 plays a role and needs further verification. Moreover, Zhou et al. found that *lncRNA PVT1* is overexpressed in osteosarcoma, reducing the survival rate of patients with osteosarcoma. *PVT1* mainly acts as a competitive endogenous RNA (ceRNA) to negatively regulate *miR-195* in osteosarcoma cells to enhance *FASN* expression, thereby promoting osteosarcoma cell migration and invasion 98.

#### Stearoyl-CoA desaturase

SCD is a major enzyme involved in the synthesis of monounsaturated FAs. Two types of SCD isoforms, SCD1 and SCD5, have been found in humans 99. Among them, SCD1 is widely expressed in tissues. The ratio between saturated FAs and unsaturated FAs is very important for cancer cells, and its changes will affect cell fluidity and protein dynamics 84. The increase in unsaturated FAs not only facilitates the proliferation and metastasis of cancer cells but also inhibits cell apoptosis. In many types of cancers, increased expression of SCD1 can lead to the proliferation and invasion of cancer cells, and inhibition of SCD1 expression can inhibit tumor progression *in vivo*, such as prostate cancer 100, bladder cancer 101, lung cancer 102 and clear cell kidney cell carcinoma 103.

The expression of *lncRNA SNHG16* is upregulated in colorectal cancer and is regulated by c-Myc. The upregulation of *SNHG16* was confirmed after c-Myc overexpression. To further clarify the potential molecular mechanism of *SNHG16*, genome-wide transcription profiling was performed after knocking down *SNHG16*. Next, Ingenuity Pathway Analysis (IPA) was used to determine that knocking out *SNHG16* can affect the expression of lipid metabolism-related genes. The study discovered that the miRNA bound by *SNHG16* cotargeted the 3'-UTR of *SCD* mRNA. Therefore, *SNHG16* may upregulate the expression of *SCD* through a ceRNA mechanism to promote the proliferation and migration of colorectal cancer cells 104. In addition, UC (ultraconserved, UC) RNA is also a long noncoding RNA. After overexpression of *uc.372* in liver cancer HepG2 cells, *FASN*, *ACC*, *SCD1* and *CD36* were all upregulated. To further study the molecular mechanism of *uc.372* regulating the expression of *ACC*, *FAS*, *SCD1* and *CD36*. Microarray analysis showed that *pri-miR-195* and *pri-miR-4668* were complementary to the ultraconserved region of *uc.372*. Overexpression of *uc.372* can inhibit the maturation of *miR-195* and *miR-4668* by specifically binding to *pri-miR-195* and *pri-miR-4668* 105. Based on previous research, *ACC* and *FAS* are the target genes of *miR-195* 106. Experimental prediction and verification proved that *SCD1* and *CD36* are the target genes of *miR-4668* 105. The overexpression of *uc.372* reduced the suppression of *ACC* and the expression of *FASN* by *miR-195* and the suppression of *SCD1* and the expression of *CD36* by *miR4668*. Therefore, it can be concluded that *uc.372* can upregulate *ACC, FASN*, *SCD1* and *CD36* through the *pri-miR-195/miR-195* and *pri-miR-4668/miR-4668* signal axes to drive fat accumulation in HepG2 cells 105. Moreover, *lncRNA UPAT* is necessary for the tumorigenicity of colon cancer cells. Through a series of experiments to identify proteins that may be related to *UPAT*, researchers found that *UPAT* can interfere with the ubiquitination and degradation of UHRF1 in colon tumors through the proteasome, thereby maintaining the stability of UHRF1. Knockout of *UPAT* or *UHRF*1 reduces the expression of *SCD1*, but *UHRF1* has nothing to do with the *SCD1* promoter region. Therefore, it is speculated that *SCD1* is not a direct target of *UHRF1* but is indirectly upregulated downstream of *UHRF1* and *UPAT*. The effect of lncRNA UPAT on SCD1 needs further study 107.

### LncRNA regulates the exogenous uptake of fatty acids

Although the *de novo* synthesis of FA in cancer occupies a major position in the synthesis of macromolecules, some FAs can also be transported by certain proteins 64, and circulating FAs can be absorbed and utilized to provide support for the survival and development of cancer cells 84. Therefore, transport proteins such as fatty acid translocase (FAT/CD36) and fatty acid-binding protein (FATP) in the FA uptake pathway may become potential targets for cancer treatment.

#### Fatty acid translocase

FAT/CD36 is a widely expressed transmembrane protein that mediates the uptake of FAs. It can also bind to carnitine palmityl transferase 1 (CPT1) in fatty acid oxidation (FAO), thus promoting FAO and providing sufficient energy for the rapid proliferation and development of cancer cells 108. However, few studies have demonstrated the influence of noncoding RNAs on FAT/CD36 regulation in cancer. Only studies have shown the effect of noncoding RNAs on FAT/CD36 in atherosclerosis.

CD36 is a key mediator of macrophage phagocytosis of oxidized low-density lipoprotein (oxLDL) in atherosclerosis 109. In recent years, studies have found that macrophages can change their phenotypes according to changes in the microenvironment and thus have diverse functions. The two main macrophage phenotypes are classically activated macrophages (M1) and selectively activated macrophages (M2) 110. The M1 phenotype has obvious proinflammatory and antitumor activities, while the M2 phenotype is an anti-inflammatory phenotype with protumor activity 111. Although both M1 and M2 macrophages are present in atherosclerosis, M1 macrophages are a dominant phenotype associated with plaque progression 112. M1 macrophages form foam cells after phagocytosing oxLDL. Foam cells then secrete proinflammatory mediators to further aggravate the production of unstable atherosclerotic plaques 113. *LncRNA MALAT1* can accumulate β-catenin on the CD36 promoter-binding site in macrophages, promote CD36 transcription and lipid uptake 114, and ultimately accelerate the occurrence of atherosclerosis. *LncRNA PELATON* is rich in unstable atherosclerosis, especially in the areas where the nuclei of macrophages gather. RNA sequencing found that there was a strong positive correlation between *PELATON* and *CD36*. With the decrease in *PELATON, CD36* expression was significantly reduced, leading to marked reductions in macrophage phagocytosis and lipid absorption, which inhibited plaque progression 115. Apart from atherosclerosis, macrophages are also closely related to the poor prognosis of tumors 116. Tumor-associated macrophages (TAMs) are the main component of inflammatory cells that infiltrate cancer. In advanced cancers, most macrophages are of the M2 phenotype, which highly stimulates tumor progression 116. Therefore, whether lncRNAs affect the role of TAMs by regulating CD36 still needs to be studied.

#### Fatty acid-binding protein

Fatty acid-binding protein (FABP) is involved in FA transport and metabolism, and FABP can bind to FFAs and transport them to various organelles for further oxidation or esterification. Multiple studies have shown that FABP5 can be involved in the occurrence and development of hepatocellular carcinoma (HCC) 117, BC 118 and prostatic carcinomas 119.

Shang et al. found that *lncRNA LNMICC* was a valuable prognostic predictor of cervical cancer. Multiple regression analysis revealed that high LNMICC expression was significantly related to the BMI of cervical cancer patients. Therefore, it is speculated that *LNMICC* is related to the reprogramming of FA metabolism in cervical cancer. For further confirmation, the levels of cell-related lipids and the expression of key FA metabolism were measured at different expression levels of *LNMICC*. Researchers have revealed that the levels of cell-related lipids and the expression of key enzymes for FA metabolism in cervical cancers with different *LNMICC* levels are different. Next, the researchers further studied how *LNMICC* exerts its biological function to reprogram FA metabolism. Based on bioinformatics analysis, *LNMICC* is located upstream of the *FABP5* gene, and the mRNA levels of *LNMICC* and *FABP5* are positively correlated. A series of experiments discovered that *LNMICC* can target the *FABP5* promoter region by recruiting *NPM1* to interact directly with *LNMICC*, thereby enhancing the transcription of *FABP5*. Finally, combined *in vivo* and *in vitro* biological function experiments show that *LNMICC* can promote lymph node metastasis and epithelial-mesenchymal transition (EMT) by directly regulating FABP5-mediated FA metabolic reprogramming 120.

### LncRNAs regulate the oxidation of fatty acids

Fatty acid oxidation (FAO) is an important catabolic process in which living organisms use FAs as energy sources. FAs must be activated before oxidation. FAs are activated under the action of ACS to generate acyl-CoA. Acyl-CoA enters the mitochondria under the action of carnitine palmityl transferase (CPT) and is oxidized and decomposed to produce acetyl-CoA, FADH2 and NADH. Acetyl-CoA enters the TCA cycle and is completely oxidized to produce ATP, while NADH and FADH2 enter the electron transport chain to produce ATP. According to reports, many types of cancers show high FAO activity, such as GC 121, BC 122, glioma 123 and acute myeloid leukemia (AML) 124. FAO can promote tumor development by increasing the production of ATP. NADPH produced by acetyl-CoA in the TCA cycle provides cancer cells with a redox ability to resist oxidative stress 125. Therefore, targeting key enzymes in FAO may hopefully provide an effective method for cancer treatment.

#### Acyl-CoA synthetase long chain family member

FAs can be catabolized to acetyl-CoA to promote the production of ATP or serve as the raw material for the synthesis of TAG, PL and CE 63, 126. However, these two distinct pathways require a common initial step, which is referred to as FA activation. ACS is an enzyme necessary for the activation of FAs. Among them, acyl-CoA synthetase long-chain family members (ACSLs) are the most critical enzymes responsible for the activation of the most abundant long-chain FA metabolism in mammalian cells. ACSLs include ACSL1, ACSL3, ACSL4, ACSL5 and ACSL6 127. Different members of ACSLs play various roles in cancer, and their expression varies in different cancers. For example, ACSL4 is highly expressed in colon adenocarcinoma 128, but it inhibits tumors in GC 129.

According to reports, lncRNAs also have the potential to regulate ACSLs. For example, *lncRNA SNHG7* plays a carcinogenic effect in thyroid cancer (TC). After *SNHG7* is knocked down, the mRNA and protein levels of *ACSL1* are suppressed. Cell proliferation and migration experiments revealed that the increase in ACSL1 can reverse the inhibitory effects of *SNHG7* on cell proliferation and migration when *SNHG7* is depleted. In addition, bioinformatics analysis found that *miR-449a* can act as a miRNA that simultaneously binds *SNHG7* and *ACSL1*. To analyze the role of* miR-499a* in the regulation of *ACSL1* by *SNHG7*, researchers conducted RIP and luciferase reporter gene analyses. The experiment concluded that *SNHG7* can be used as a sponge for *miR-449a*, thereby increasing *ACSL1* in TC cells and promoting the proliferation and migration of TC cells 130. Additionally, lncRNA *NEAT1* is highly expressed in docetaxel-resistant prostate cancer patients and cell lines. The CCK-8 experiment confirmed that after knocking out *NEAT1*, the IC50 value of docetaxel on prostate cancer cells decreased significantly. Compared with docetaxel-treated parental cells, NEAT1 knockdown promoted the sensitivity of cells to docetaxel and reduced cell proliferation and invasion. To study the potential molecular mechanism of *NEAT1* in prostate cancer, bioinformatics predictions and experiments proved that *miR-34a-5p* and *miR-204-5p* are potential targets of *NEAT1* and are inhibited by *NEAT1*. Next, the downstream targets of *miR-34a-5p* and *miR-204-5p* were predicted and verified to be *ACSL4*. *miR-34a-5p* and *miR-204-5p* can inhibit the expression of *ACSL4* by targeting the 3'-UTR of *ACSL4* and then reduce the ability of prostate cancer cells to be resistant to docetaxel. In summary, *NEAT1* can enhance the expression of *ACSL4* by sponging *miR-34a-5p* and *miR-204-5p*, thereby promoting docetaxel resistance in prostate cancer cells and accelerating the progression of prostate cancer 131. In addition, studies have shown that *lncRNA HULC* can regulate abnormal lipid metabolism in the body through the miR-9/PPARA/ACSL1/signaling pathway 132, increase the accumulation of TG and CE in HCC tissues, and promote the growth and development of HCC. The mechanism of this signaling pathway will be explained in detail below.

#### Carnitine palmityl transferase

At present, it is known that FAO plays a role in inducing cancer cell metastasis and chemotherapy resistance and improving the stemness of cancer cells 65, 121, 133-135. Among them, CPT1, as the rate-limiting enzyme of FAO, provides the first and rate-limiting steps of FA transport to mitochondria for oxidation. CPT1 can directly control the production of ATP and NADPH in the FAO process, which constitutes an important part of cancer metabolic adaptation 125. Therefore, fully exploring the molecular mechanism of lncRNA regulating CPTI can identify a new therapeutic window in cancer treatment intervention.

Metastasis-associated in colon cancer-1 (MACC1) is a transcriptional regulator of EMT. *MACC1* is upregulated in various tumors and enhances cell proliferation, invasion and chemotherapy resistance 136-140. *MACC1-AS1* is the cognate antisense lncRNA of *MACC1*. Recent studies have shown that *MACC1-AS1* can be induced to be expressed by mesenchymal stem cells (MSCs) 121. In previous reports, MSCs secreted TGF-β1, a key cytokine. TGF-β exerts cellular effects by binding to TGFβ receptors (TGFβR-I and TGFβR-II) and then activates the downstream SMAD family 141. In a recent study, it was found that when MSCs are cocultured with GC cell lines, TGF-β1 secreted by MSCs can be directly combined with TGFβR-I and TGFβR-II in GC cells to activate SMAD2 and SMAD3 and then induce the upregulation of *MACC1-AS1* in GC cells. The upregulation of *MACC1-AS1* activates the FAO pathway in GC cells, and the expression levels of FAO-related enzymes (CPT1 and ACS) are significantly increased, which promotes stemness and chemoresistance in GC. In addition, to clarify the potential mechanism of *MACC1-AS1* on FAO-dependent dryness and chemical resistance, it was found that *miR-145-5p* is located downstream of *MACC1-AS1* based on the prediction of the LncRNASNP database and experimental verification, and *MACC1-AS1* can directly bind to *miR-145-5p* and inhibit the expression of *miR-145-5p. miR-145-5p* partially reversed the promoting effect of *MACC1-AS1* on the expression of *CPT1* and stem genes and inhibited the occurrence of drug resistance and FAO 121. In summary, the TGF-β1/MACC1-AS1/miR-145-5p/CPT1 signaling axis contributes to FAO-dependent stem and chemoresistance, indicating that targeting this signaling pathway may be a potential strategy to inhibit stem cells and chemoresistance induced by MSCs. LncRNAs termed *HCP5* could be induced in GC cells by coculture with MSCs and were reported to play an important role in elevating GC cancer cell stem properties and chemoresistance and in predicting a poor prognosis. The pull-down assay showed that *miR-3619-5p* can be pulled down by *HCP5* in GC cells, indicating that there is an interaction between *HCP5* and *miR-3619-5p*. Then, in the biological function experiment, the effects of *HCP5* and *miR-3619-5p* were opposite. *HCP5* can exert a sponge effect on *miR-3619-5p* to promote the activity of CPT1 in GC cells. To further explore the molecular mechanism of this, KEGG analysis revealed that *miR-3619-5p* was significantly related to the AMPK pathway. The most significantly downregulated gene after *miR-3619-5p* overexpression was PPARG coactivator 1α (PPARGC1A), a key regulator of the AMPK pathway 142. It was previously reported that PGC1α, the protein product of *PPARGC1A*, is a transcriptional coactivator responsible for lipid metabolism 143. This study revealed that PGC1α can form a transcription complex with CEBPB to activate the transcription of *CPT1* and ultimately promote FAO in GC cells. Therefore, the FAO of GC cells can be driven by the *HCP5/miR-3619-5p/PPARGC1A*/PGC1α/*CPT1* axis in GC cells activated by MSCs and ultimately promote the chemoresistance and stemness of GC cells 142.

In addition, another lncRNA induced by MSCs is *lncRNA AGAP2-AS1*, which was significantly upregulated in BC cells cocultured with MSCs. Bioinformatics analysis showed that the target gene of *AGAP2-AS1* was enriched in FAO metabolism. Then, experiments verified that *AGAP2-AS1* mediated stemness and trastuzumab resistance by targeting *CPT1* and *ACS*. Further research on the regulatory mechanism of *AGAP2-AS1* found that the interaction between* AGAP2-AS1* and *CPT1* is achieved in two ways. First, *AGAP2-AS1* can interact with *Hur* to produce *AGAP2-AS1-Hur* and then directly bind to *CPT1* mRNA to improve the stability of *CPT1*. Second, *AGAP2-AS1* inhibited the expression of *miR-15a-5p* through sponge action to increase *CPT1* mRNA. Under the combined actions of these two pathways, *AGAP2-AS1* can upregulate the expression of *CPT1* at the mRNA level, and the FAO of BC cells can be promoted, thereby mediating the characteristics of cancer stem cells and the resistance of trastuzumab 122. In addition, it has been reported that *lncRNA NEAT1* can competitively bind to *miR-107* in BC cells, indirectly inhibit the inhibitory effect of *miR-107* on *CPT1*, promote FA oxidation, and provide ATP to regulate the growth and metastasis of BC 144.

## LncRNAs are closely related to abnormal triglyceride metabolism in cancer

TG is a key energy source composed of FFA. When there is too much FA, most of the FA can be connected to the glycerol backbone to form TG, which is then stored in cytoplasmic lipid droplets (LDs) 145. This storage itself is not disease-causing, but TG, as a substance for storing FA, can release FA at any time. This ready availability may provide cancer with much energy and promote tumor proliferation and metastasis 64. The accumulation of lipids can also cause lipotoxicity, inducing apoptosis 146. Therefore, increasing FA storage and inhibiting FA release may also be cancer suppression strategies 147 (Figure 1 and Table 1).

### LncRNAs regulate the synthesis of triglyceride

First, FA is activated into fatty acyl-CoA (FA-CoA), and FA-CoA forms lysophosphatidic acid (LPA) under the action of glycerol 3-phosphate acyltransferase (GPAT). LPA is converted into phosphatidic acid (PA) under the action of acylglycerophosphate acyltransferase (AGPAT). Then, phosphatidic acid phosphohydrolase (PAP or lipin) removes the phosphate group from PA to form diacylglycerol (DG). Finally, diacylglycerol acyltransferase (DGAT) esterifies DG and FA-CoA to TG 148, 149. Therefore, by reducing FA utilization and increasing its storage as TG, tumor progression can be inhibited. For instance, *lncRNA SPRY4-IT1* is highly expressed in melanoma, and the protein related to *SPRY4-IT1* is identified as lipin-2 by mass spectrometry analysis. *Lipin-2* can be used as the direct target of *lncRNA SPRY4-IT1*. Downregulation of *SPRY4-IT1* resulted in the enhancement of *lipin-2* mRNA, protein and enzyme activities. Due to the effective conversion of DAG mediated by lipin-2 to TAG, both the *DGAT* mRNA expression level and the TG content increased. Based on these results, it is speculated that *SPRY4-IT1* knockdown may induce the apoptosis of melanoma cells through lipin-2-mediated lipotoxicity, but this speculation needs further experimental verification 150.

In addition to directly regulating the enzymes in the TG synthesis pathway, lncRNAs can also indirectly regulate the synthesis of FA and TG by regulating SREBP-1c to achieve lipid metabolism reprogramming. SREBP-1c mainly activates the transcription of multiple genes, such as *ACLY*, *ACC*, *FAS*, *SCD-1*, and *GPAT*. The enzymes encoded by these genes can participate in the synthesis of FAs and TG 151. Therefore, targeting SREBP-1c can effectively inhibit the production of lipids and prevent the proliferation of cancer cells. Li et al. found that overexpression of *lncRNA HR1* in Huh7 liver cancer cells can inhibit the phosphorylation of AKT. AKT acts as an upstream regulator of Forkhead Box O1 (FoxO1), and inhibition of AKT phosphorylation reduces the nuclear translocation of FoxO1, which causes a large accumulation of FoxO1 in the nucleus 152. In previous reports, FoxO1 was generally regarded as a tumor suppressor that can antagonize the combination of the LXRα/RXR heterodimer and LXR elements (LXREs) in the promoter region of *SPEBP-1c*, thereby downregulating the transcription of SREBP-1c 153. In summary, *lncHR1* may inhibit the accumulation of TG in liver cancer cells through the AKT/FoxO1/SREBP-1c pathway and ultimately may reduce the energy supply of cancer cells to inhibit tumor progression 152.

### LncRNA regulates the degradation of triglyceride

FA can be stored in the form of TG. When needed, each TG molecule can be sequentially catalyzed by triglyceride lipase (ATGL), hormone-sensitive lipase (HSL) and monoacylglycerol lipase (MAGL) to release three FAs. ATGL is usually considered the key enzyme for the release of FAs from TG stores 154, 155. High levels of ATGL have been discovered in a variety of cancers, such as BC 156 and lung cancer 157. Liu et al. found that the expression of ATGL in HCC tissues is upregulated, which is related to poor prognosis. Co-lncRNA software screening and assay identification showed that *lncRNA NEAT1* and ATGL were positively correlated in HCC tissues. To explore the molecular mechanism of NEAT1/ATGL, researchers used a series of bioinformatics methods to determine whether there is a potential miRNA to regulate ATGL. Studies have found that *miR-124-3p* can directly bind *ATGL* to inhibit the expression of *ATGL*. However, the interaction between *NEAT1* and *ATGL* may occupy the binding site of *miRNA-124-3p* and *ATGL*, weakening the inhibitory effect of *miR-124-3p* on *ATGL* 158. In addition, it has been proven that FA is the main physiological ligand that activates the known oncogene *PPARα* in liver cancer 159. NEAT1 abnormally regulates lipolysis, which leads to an increase in FA, which eventually increases the expression of PPARα, thereby promoting the growth and reproduction of HCC cells 158. These results indicate that NEAT1 can mediate the growth of HCC cells through miR-124-3p/ATGL/DAG + FA/PPARα signaling.

## LncRNAs control phospholipid metabolism in cancer

Phospholipids are the key components of cell membranes and can be divided into two categories: glycerophospholipids and sphingomyelin. Phospholipids play important roles in cells, such as chemical energy storage, cell signal transmission, and cell-cell interactions 160. Since uncontrolled cell proliferation in cancer requires sufficient energy and cell structural units, the phospholipids in cancer cells need to be actively biosynthesized so that phospholipids can perform the abovementioned functions and participate in the development of cancer (Figure 1 and Table 1).

### Sphingosine kinases

Sphingosine kinases (SphKs) are key regulatory enzymes that catalyze the formation of sphingosine-1-phosphate (S1P). SphK1 is a cytoplasmic protein. Once Sphk1 is activated by various extracellular signal transduction pathways, it is phosphorylated by ERK1/2 and transferred to the plasma membrane to phosphorylate sphingosine to form S1P 161. Conversely, SphK2 is mainly located in the nucleus and part of the mitochondria, and S1P can also be produced at these sites 162, 163. Currently, S1P is involved in regulating cell growth, proliferation, survival, migration and angiogenesis 164. Therefore, SphKs may be an important tumorigenesis regulator. Further understanding of the molecular mechanisms regulating SphK expression may reveal new directions for therapeutic strategies. It has been found that the abnormal expression of SphKs is associated with the development and poor prognosis of many cancers 165-168. There are a variety of lncRNAs that can participate in various types of cancer-related biological abnormalities by regulating the expression of SphKs.

In osteosarcoma, Anna et al. found that *lncRNA Khps1* is an antisense RNA that can activate the transcription of *SphK1*. Therefore, researchers have studied the molecular mechanism by which *Khps1* regulates the expression of *SphK1*. *SphK1* contains two putative triplex-forming regions (TFRs) upstream of the transcription start site of its subtype B (SphK1-B). *Khps1* can directly interact with *TFR2* to form a purine-rich RNA-DNA triplet through Hoogsteen hydrogen bond base pairing, which is anchored to the *SphK1-B* promoter. *Khps1* linked to *SphK1-B* can interact with histone acetyltransferase p300/CBP, which modifies histone acetylation to create an open chromatin structure. The change in chromatin structure promotes the binding of the *SphK1-B* upstream transcription factor E2F1 to the *SphK1-B* promoter to activate *SphK1* transcription. Interestingly, the DNA fragment containing the *Khps1* promoter also has a binding site for the transcription factor E2F1. Combined with kinetic data analysis, E2F1 induces *Khps1* transcription before promoting *SphK1-B* mRNA upregulation. Therefore, elevated levels of E2F1 will induce the transcription of *Khps1.* Then, *Khps1* changes the chromatin structure of *SphK1-B* to promote the combination of E2F1 and the *SphK1-B* promoter to activate *SphK1-B* transcription and ultimately promote the proliferation of osteosarcoma cells and limit cell apoptosis 169. In HCC, Zhan et al. found that *lncRNA HULC* can increase the expression levels of *SphK1* mRNA and protein and promote the angiogenesis of HCC. Studies on the transcriptional regulation of *SphK1* by* HULC* showed that *E2F1* was involved in the activation of *SphK1* transcription by *HULC*. To study the relationship between *HULC* and *E2F1*, the 3'UTR of *E2F1* mRNA was analyzed. Studies have demonstrated that *miR-107* inhibits the expression of *E2F1* by targeting the 3'UTR of *E2F1* mRNA. Based on the fact that lncRNAs can be used as ceRNAs to regulate miRNAs, experiments have observed that *HULC* can act as a sponge to inhibit *miR-107* to upregulate *E21F*, thereby promoting the expression of *Sphk1*. Therefore, these results indicate that *HULC* sponges *miR-107* and activates the *SphK1* promoter under the action of the transcription factor E2F1, induces the upregulation of *SphK1*, and finally stimulates the angiogenesis of HCC 170. In addition, *LINC00460* is significantly upregulated in colorectal cancer. miRcode software was used to predict the potential target of *LINC00460.* The results show that *miR-613* is a direct potential target of *LINC00460. LINC00460* can be used as a ceRNA sponge for *miR-613* to increase the expression of the downstream target gene *Sphk1* of *miR-613*, thereby promoting the proliferation, migration and invasion of colorectal cancer cells 171.

In addition to SphK1, lncRNAs can also regulate Sphk2 to participate in tumorigenesis and development. In papillary thyroid carcinoma (PTC), the expression of *LINC00460* is upregulated, which is positively correlated with patients with advanced lymph node metastasis. It was further discovered that *LINC00460* can be used as a ceRNA to regulate the expression of *SphK2* by sponging *miR-613* and promoting cell proliferation, migration and invasion 172. Another study reported that *LINC00520* sponged *miR-577* in PTC cells with a similar mechanism to positively regulate *Sphk2* expression, leading to increased PTC cell proliferation, migration, invasion and apoptosis 173.

### Phospholipase

Phospholipases are enzymes that are involved in the hydrolysis of acyl and phosphate esters of phospholipids. They are important mediators of intracellular and intercellular signaling. Lipid mediators generated by phospholipases regulate multiple cellular processes that can promote cancer development 174.

Phospholipase D (PLD) hydrolyzes phosphatidylcholine (PC) to PA, a signaling lipid, which regulates cell growth and cancer progression through effects on mTOR and PKB/Akt 175. Thus, PLD expression is increased in cancers, where it correlates with tumor size and prognosis 176, 177. *LINC00511* is upregulated in cervical cancers and could promote *PLD1* expression by enriching *RXRA* to the promoter region of *PLD1* and activating *PLD1* promoter activity, thus enhancing proliferation and inhibiting the autophagy and apoptosis of cervical cancer cells 178.

Phospholipase A2 (PLA2) catalyzes the hydrolysis of phosphatidic acid, resulting in the production of FFAs and lysophospholipid 1 179. There are dozens of secreted PLA2 forms called secreted phospholipase A2 (sPLA2). sPLA2 has oncogenic functions in cancer biology and is usually upregulated in certain types of cancers, such as triple-negative BC 180, NSCLC 181 and ovarian cancer 182. Studies have found that *lncRNA SLNCR1* is highly expressed in NSCLC and may regulate the migration, invasion, and dryness of NSCLC by interacting with sPLA2 181. However, further research is needed to determine the mechanisms involved in this process.

## Lipid metabolism influences lncRNA expression

**Figure 2:**
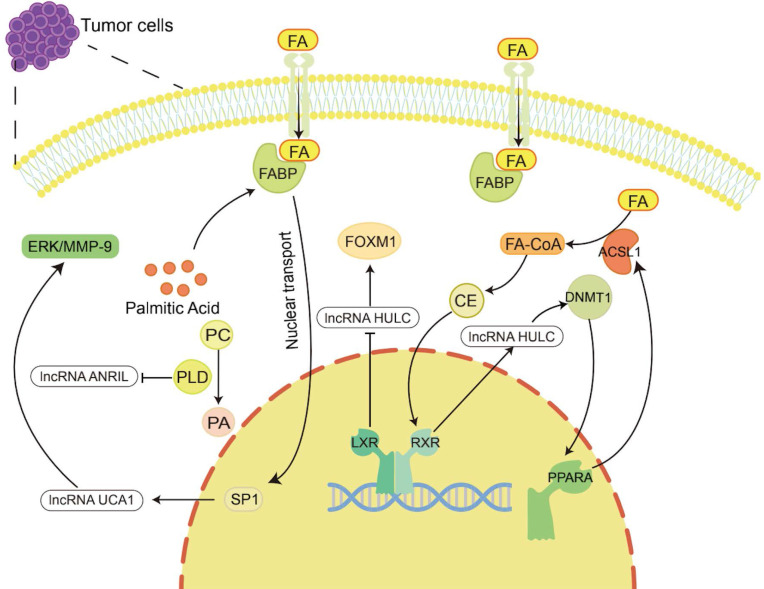
** Lipid metabolism regulates the occurrence and development of tumors by regulating lncRNAs.** Lipid metabolites and enzymes involved in lipid metabolism can in turn regulate the expression of lncRNAs to participate in tumor progression. For example, cholesterol regulates *lncRNA HULC* to promote liver cancer progression, palmitic acid regulates *lncRNA UCA1* to promote gastric cancer metastasis, and PLD regulates *lncRNA ANRIL* to exert an anti-large cell lung cancer effect.

The products of lipid metabolism are necessary to support cell survival and development. In the above, lncRNAs affect the expression of metabolites by regulating various enzymes involved in cholesterol metabolism, FA metabolism, TG metabolism, and PL metabolism, thereby affecting the development of cancer. However, lipid metabolites and enzymes involved in lipid metabolism also have a regulatory effect on the expression of lncRNAs, which affects the occurrence and development of tumors (Figure 2 and Table 1).

For example, Cui et al. showed that *lncRNA HULC* expression was upregulated by cholesterol through a positive feedback loop involving the retinoid receptor *RXRA*, which activated the *HULC* promoter 132. *HULC* exerts its effect by upregulating the expression of methylase DNA (cytosin-5-)-methyltransferase 1 (DNMT1) and thereby inhibiting the expression of *miR-9* by eliciting methylation of CpG islands in the *miR-9* promoter, preventing *miR-9* from targeting the 3'UTR of *PPARA*. As a consequence, the transcription level of *PPARA* in hepatoma cells was upregulated. Given that *ACSL1* is activated by the transcription factor PPARA 183, overexpression of *ACSL1* further increases the uptake of FAs in HCC cells 184, and its product cholesterol upregulates *HULC* by activating *RXRA*. Hence, forming a positive feedback loop to upregulate the level of *HULC* in hepatoma cells leads to abnormal lipid metabolism and accelerates the occurrence of HCC 132. LXRs are cholesterol sensors 51. He et al. found that *LXRα* can bind to the *lncRNA HULC* promoter region and inhibit the transcriptional activity of the *HULC* gene promoter. *HULC* can inhibit *miR-134-5p* targeting the 3'-UTR of *FOXM1* to upregulate the expression of *FOXM1* 185. In previous studies, FOXM1 has been proven to play a promoting role in a variety of tumors, including HCC 186-188. Therefore, *LXR* can inhibit the growth of HCC cells by reducing *HULC*, increasing *miR-134-5p* and reducing *FOXM1* 185.

Palmitic acid is the most common saturated FA and can be synthesized endogenously *de novo* or ingested exogenously. It maintains a certain concentration under physiological conditions, maintains membrane stability and participates in protein transport 189. It has been reported that palmitic acid can promote the nuclear transport of FABP5, thereby increasing the nucleoprotein level of SP1 190. SP1 can bind to the promoter of *lncRNA UCA1* to increase the expression of *UCA1* 191. Then, *UCA1* can regulate GRK2 protein stability by promoting the Cbl-c-mediated ubiquitination and degradation of GRK2, thereby activating the ERK-MMP9 pathway to increase GC cell metastasis 192. In addition, PLD is an important enzyme involved in regulating phospholipid metabolism. It has been reported that in large cell lung cancer H460 cells, inhibiting PLD expression can stimulate the upregulation of lncRNA ANRIL, promote cell apoptosis, and produce antitumor effects 193.

In addition, Li et al. found that endogenously synthesized n-3 polyunsaturated FAs in fat-1 mice decrease the mammary cancer risk of female offspring by regulating the expression of lncRNAs (53 upregulated and 45 downregulated) 194.

## LncRNAs act as regulators of ferroptosis

**Figure 3:**
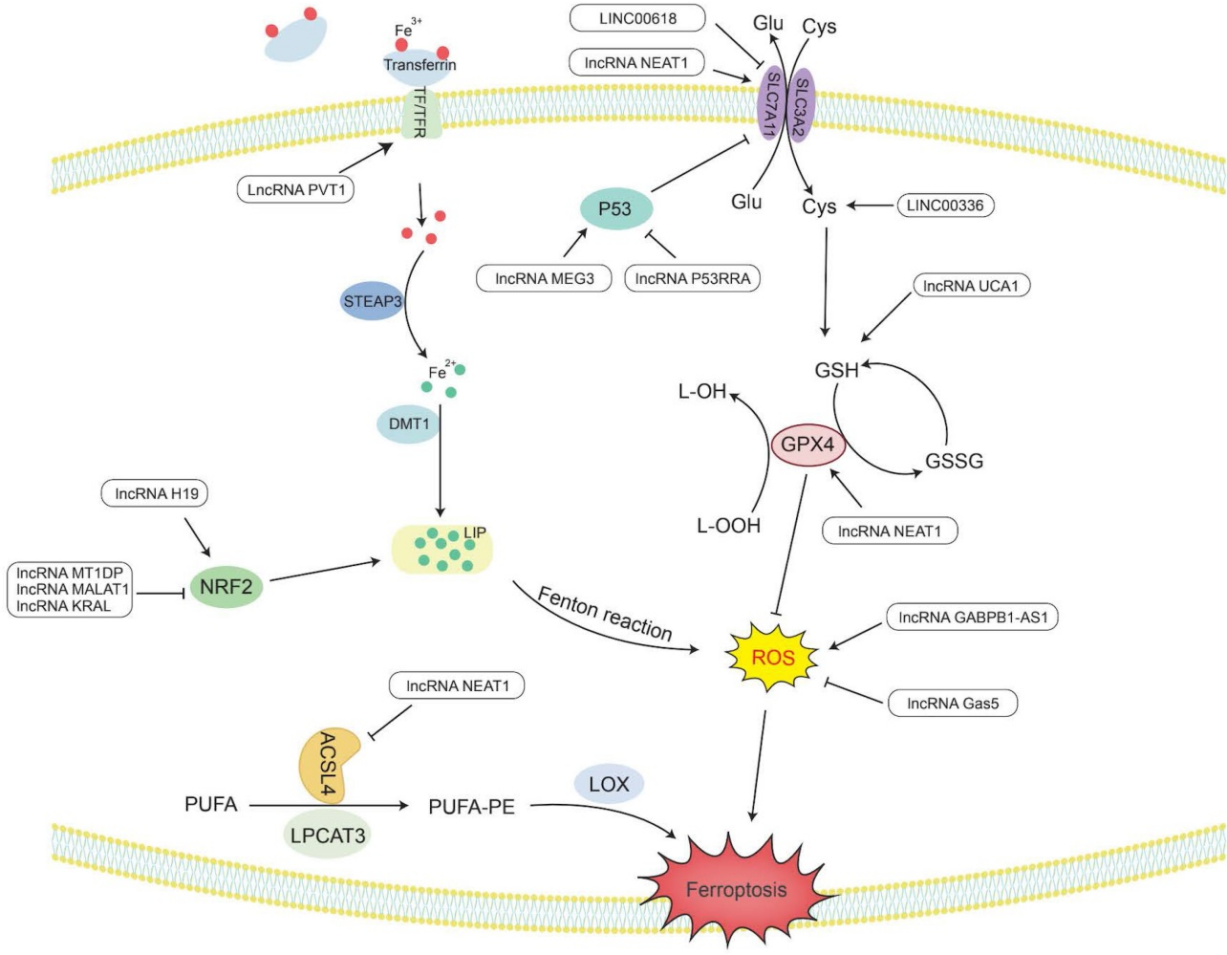
** The metabolic pathway of lncRNAs regulating ferroptosis.** Lipid peroxides play a key role in initiating ferroptosis. LncRNAs participate in the occurrence of ferroptosis in tumors by regulating related genes involved in lipid peroxide accumulation.

Ferroptosis is a novel type of special cell programmed death that is mainly caused by lipid peroxidation induced by ROS, resulting in lipid peroxide accumulation 8. In particular, the peroxidation of PUFAs can lead to the formation of lipid hydrogen peroxide (LOOH), which is cleaved under the action of active Fe^3+^ to form lipid free radicals that are highly reactive and toxic to membranes and cells. These lipid free radicals can extract electrons from adjacent PUFAs, thereby continuing to trigger new LPO and oxidative damage 9. The inactivation of GPX4 depends on glutathione (GSH), lipid peroxide cannot be catalyzed and reduced by GPX4, and Fe^2+^-oxidized lipids produce a large amount of ROS by the Fenton reaction, all of which promote ferroptosis 195. Therefore, studying the mechanism by which lncRNAs affect ferroptosis would provide new ideas for the treatment of cancer (Figure 3 and Table 2).

### LncRNAs regulate the activity of GPX4 in ferroptosis

GPX4 is a GSH-dependent enzyme 196, and its antioxidant function requires the use of GSH as a substrate 197, thereby reducing L-OOH to the corresponding nontoxic alcohol, losing its peroxide activity 195, hence reducing ROS and subsequently preventing the occurrence of ferroptosis.

LncRNAs play an indispensable role in regulating ferroptosis. *LncRNA UCA1* sponges *miR-16* to upregulate the expression of glutaminase 2 (GLS2) in bladder cancer 198. GLS2 can catalyze the conversion of glutamine into glutamic acid and further synthesize GSH. When glutamine is metabolized, it enters the tricarboxylic acid cycle to produce a large amount of nicotinamide adenine dinucleotide phosphate (NADPH) 199. This NADPH is required when glutathione reductase (GR) reduces oxidized glutathione (GSSG) to GSH 200. Therefore, *UCA1* can upregulate the expression of GSH and NADPH, making GPX4 play an antioxidant role to reduce the production of ROS and inhibit ferroptosis.

It has been reported that in the human ACSL family, only ACSL4 is related to ferroptosis 201. Saturated membrane lipids are not sensitive to peroxidation, but ACSL4 can enrich cell membranes with polyunsaturated omega-6 FAs 201, 202. Therefore, the upregulation of ACSL4 promotes the occurrence of LPO and the sensitivity of cells to ferroptosis 203. For example, *lncRNA NEAT1* can directly bind to ACSL4. Overexpression of *NEAT1* can inhibit the expression of ACSL4, increase the expression of SLC7A11 and GPX4, and reduce the content of regulatory iron in cells. Finally, the occurrence of ferroptosis is decreased, and cell apoptosis is inhibited 204.

In addition, nuclear factor erythroid 2-related factor 2 (Nrf2) acts as an antioxidant transcription factor and activates the expression of downstream antioxidant factors. Nrf2 can upregulate genes in the pentose phosphate pathway to produce NADPH, which plays an antioxidant role. Nrf2 can increase the transcription of genes involved in the synthesis of GSH and GPX family genes and increase the activity of GPX4 in ferroptosis to exert antioxidant effects 205. In ovarian cancer, *lncRNA H19* can target Nrf2 to regulate genes related to GSH synthesis. The increase in GSH synthesis enhances the antioxidant effect and promotes cisplatin resistance 206. In addition, Keap1 (Kelch-like ch-associated protein 1) is a protein that can target the ubiquitination of Nrf1 and accelerate the degradation of Nrf1 in the proteasome 207. Therefore, there are still some lncRNAs that affect the generation of ROS by regulating the Keap1/Nrf1 pathway. For example, *lncRNA KRAL* can act as a ceRNA of *miR-141* in HCC to upregulate the expression of *Keap1*, thereby downregulating the expression of Nrf1, weakening the expression of downstream genes in the Nrf2 pathway, and increasing the sensitivity of cancer cells to 5-fluorouracil 207. Overexpression of *lncRNA MALAT1* in multiple myeloma can upregulate Keap1 expression to target Nrf1, causing it to undergo proteasome degradation, leading to the occurrence of oxidative stress 208.

### LncRNAs regulate Fe^2+^ levels in ferroptosis

Maintaining a balance of intracellular Fe levels is essential for cell survival. Fe^3+^ needs to be taken up into cells under the combined action of transferrin (TF) and transferrin receptor (TFR) 199. Then, Fe^3+^ is oxidized to Fe^2+^ under the redox effect of the six-transmembrane epithelial antigen of prostate 3 (STEAP3) and is ultimately stored in the labile iron pool (LIP) with redox activity and ferritin 209. In ferroptosis, Fe^2+^ can provide electrons to oxygen through the Fenton reaction to form ROS 210, which catalyzes the formation of lipid radicals that are highly reactive with biomolecules 195, 211.

*LncRNA PVT1* is upregulated in HCC and acts as a sponge for *miR-150* to regulate the expression of *HIG2*, the downstream target gene of miR-150 212. *HIG2*, as a hypoxia-inducing gene, has been determined to be involved in ferroptosis 213. The detection of iron homeostasis-related protein expression and Fe uptake showed that knocking down PVT1 significantly reduced the expression of TFR1 and increased the expression of ferritin light chain (FTL), ultimately reducing the uptake of Fe and regulating ferroptosis 212.

In addition, Nrf2 can increase the transcription level of ferroportin 1 (FPN1) to increase the export of intracellular iron to the outside of the cell and reduce the level of Fe^2+^ in the intracellular LIP. Nrf2 can also induce the upregulation of ferritin gene transcription and store excess iron in cells 205. In NSCLC cells, *lncRNA MT1DP* inhibits the expression of *Nrf2* by stabilizing *miR-365a-3p*, increases intracellular iron levels, and increases oxidative stress-induced hypertrophy caused by ROS 214.

### LncRNAs regulate cysteine metabolism in ferroptosis

SLC7A11 is a plasma membrane transporter that can take extracellular cysteine into cells and transfer intracellular glutamate to the outside of the cell. The increase in intracellular cysteine content increases the synthesis of GSH and promotes GPX4 to exert its antioxidant effect 215.

*LncRNA P53RRA* has been shown to be mainly located in the cytoplasm and is silent in a variety of cancers, such as NSCLC, liver cancer, colon cancer, NPC and BC. *P53RRA* can bind to Ras-GTPase activator protein binding protein 1 (G3BP1), resulting in the removal of P53 from the G3BP1 complex and increasing the retention of P53 in the nucleus 216. P53 inhibits the transcription of SLC7A11 in the nucleus and reduces the uptake of cysteine, consequently limiting the production of GSH in the cell. GSH acts as a cellular antioxidant, and its reduction renders GPX4 unable to function, which can easily lead to the occurrence of LPO, thus increasing the sensitivity of cells to ferroptosis 217. *LncRNA MEG3* was found to be underexpressed in NSCLC, and its overexpression can activate the expression of P53 218. Therefore, knocking down *MEG3* can reduce P53 and promote the synthesis of GSH, resulting in a decrease in ROS levels and reduced apoptosis 219. Another study found that *LINC00618* can attenuate the expression of lymphoid-specific helicase (LSH) by interacting with LSH and then affect the binding of LSH to the *SLC7A11* promoter to reduce the transcriptional activity of *SLC7A11* to induce ferroptosis 220. In addition, LSH can also promote the expression of ELAV-like RNA-binding protein 1 (ELAVL1) by reducing the recruitment of P53 to the *ELAVL1* promoter. *ELAVL1* can increase its expression in lung cancer by stabilizing the posttranscriptional level of *LINC00336*. *LINC00336* can increase the expression of cystathionine β-synthase (CBS) through the endogenous sponge of *miR-6852* 221. Then, CBS can enhance the transsulfuration pathway (TSP), which converts methionine to cysteine 222. Finally, glutathione is synthesized to further exert its antioxidant effect 223. Therefore, *LINC00336* can reduce lipid ROS and inhibit the occurrence of ferroptosis through the miR-6852/CBS/GSH pathway 221.

### LncRNAs regulate ROS levels through other mechanisms in ferroptosis

In addition to the above factors, lncRNAs also regulate ROS through other mechanisms. High expression of GABPB1 is associated with a poor prognosis of HCC. Erastin is an inducer of ferroptosis that can upregulate the expression of the antisense *lncRNA GABPB1-AS1* of the *GABPB1* gene, block the transcription and translation level of GABPB1, and inhibit the expression of peroxidase-5 (PRDX5). Therefore, the inhibitory effect of PRDX5 on the production of ROS by H_2_O_2_ is weakened, which ultimately inhibits the antioxidant capacity of cancer cells and increases cell death 224. *LncRNA Gas5* was found to induce cell cycle arrest in melanoma. Knockdown of *Gas5* can increase the expression of glucose 6-phosphate dehydrogenase (G6PD) and NADPH oxidase 4 (NOX4) 225. G6PD can promote the production of NADPH, making it function as a substrate for activating NOX4 226. NOX4 is a transmembrane protein that produces peroxide and/or hydrogen peroxide, and its upregulation can promote the production of ROS. Therefore, after knocking down* Gas5*, ferroptosis can occur under the synergistic effect of G6PD and NOX4 225.

Collectively, these results show that lncRNAs are important regulators of ferroptosis and lipid metabolism.

## Conclusion and prospects

A number of studies have revealed that reprogramming and ferroptosis of lipid metabolism are closely related to the occurrence, development and progression of tumors, expanding our understanding of the impact of lipids on tumor etiology.

Today, traditional treatment methods are gradually showing shortcomings, such as nonspecificity and drug resistance. Therefore, the role of lncRNAs in cancer has attracted increasing attention from researchers. In this review, we emphasized lncRNAs related to lipid metabolism, ferroptosis and cancer. Although there have been few studies on the role of lncRNAs in cancer, we can also see promising effects in the treatment of cancer from the limited existing literature. LncRNAs regulate multiple aspects of abnormal tumor lipid metabolism, thus playing a carcinogenic role in multiple aspects of tumorigenesis and affecting tumor cell proliferation, apoptosis, migration, invasion, ferroptosis and other biological processes. LncRNAs can also influence the onset of ferroptosis by regulating related genes in the ferroptosis pathway, enabling them to produce a way to kill cells that is not dependent on apoptosis. Therefore, we believe that targeting lncRNAs may serve as a viable strategy for cancer treatment, creating new opportunities for diagnostic therapeutic interventions in cancer.

However, there are still several problems that remain to be addressed. First, it is still challenging to identify the most important lncRNAs associated with specific types of tumors. Additionally, compared with protein-coding genes, the conservation of lncRNAs across different species is generally poor. Therefore, therapeutic strategies developed using *in vitro* and animal models cannot be easily extended to human applications immediately and may require further studies.

Taken together, future studies should focus on uncovering the relationships of each lncRNA in tumor lipid metabolism and ferroptosis and developing appropriate model systems to aid in the development of diagnosis, treatment and prognosis in cancer. We believe that with continuous research work, in the future, we could make full use of the advantages of lncRNAs as regulators of tumor metabolism. Great progress will be made in cancer treatment strategies based on lncRNAs to improve the quality of life of many cancer patients.

## Figures and Tables

**Table 1:** Summary of lipid metabolism-associated lncRNAs in cancer

LncRNA	Associated type of effect	Target	Influence to target	Cancer type	Reference
LncRNA SNHG16	Cholesterol synthesizes	SREBP2	Up	Pancreatic cancer	45
Lnc030	Cholesterol synthesizes	SQLE	Up	Breast cancer	47
LncRNA CASC19	Cholesterol uptake	LDLR	Up	Non-small cell lung cancer	48
LncRNA TINCR	*De novo* synthesis of fatty acids	ACLY	Up	Nasopharyngeal carcinoma	77
LncRNA FLJ22763	*De novo* synthesis of fatty acids	ACLY	Down	Gastric cancer	78
LncRNA DNAJC3-AS1	*De novo* synthesis of fatty acids	ACC/FASN	Up	Colorectal cancer	87
LncRNA CTD-2245E15. 3	*De novo* synthesis of fatty acids	ACC1	Up	Non-small cell lung cancer	85
LncRNA HAGLR	*De novo* synthesis of fatty acids	FASN	Up	Non-small cell lung cancer	94
LncRNA HOTAIR	*De novo* synthesis of fatty acids	FASN	Up	Nasopharyngeal carcinoma	96
LncRNA PVT1	*De novo* synthesis of fatty acids	FASN	Up	Osteosarcoma	98
LncRNA SNHG16	*De novo* synthesis of fatty acids	SCD	Up	Colorectal cancer	104
LncRNA uc.372	*De novo* synthesis of fatty acids	ACC/FASN/SCD1	Up	Liver cancer	105
LncRNA UPAT	*De novo* synthesis of fatty acids	SCD1	Up	Colon cancer	107
LncRNA uc.372	Exogenous uptake of fatty acids	CD36	Up	Liver cancer	105
LncRNA LNMICC	Exogenous uptake of fatty acids	FABP5	Up	Cervical cancer	120
LncRNA SNHG7	Oxidation of fatty acids	ACSL1	Up	Thyroid cancer	130
LncRNA HULC	Oxidation of fatty acids	ACSL1	Up	Hepatocellular carcinoma	132
LncRNA NEAT1	Oxidation of fatty acids	ACSL4	Up	Prostate cancer	131
LncRNA MACC1-AS1	Oxidation of fatty acids	CPT1	Up	Gastric cancer	121
LncRNA HCP5	Oxidation of fatty acids	CPT1	Up	Gastric cancer	142
LncRNA AGAP2-AS1	Oxidation of fatty acids	CPT1	Up	Breast cancer	122
LncRNA NEAT1	Oxidation of fatty acids	CPT1	Up	Breast cancer	144
LncRNA SPRY4-IT1	Synthesis of triglyceride	Lipin-2	Down	Melanoma	150
LncRNA HR1	Synthesis of triglyceride	SPEBP-1c	Down	Liver cancer	152
LncRNA NEAT1	Degradation of triglyceride	ATGL	Up	Hepatocellular carcinoma	158
LncRNA Khps1	Phospholipid metabolism	SphK1	Up	Osteosarcoma	169
LncRNA HULC	Phospholipid metabolism	SphK1	Up	Hepatocellular carcinoma	170
LINC00460	Phospholipid metabolism	SphK1	Up	Colorectal cancer	171
LINC00460	Phospholipid metabolism	SphK2	Up	Papillary thyroid carcinoma	172
LINC00520	Phospholipid metabolism	SphK2	Up	Papillary thyroid carcinoma	173
LINC00511	Phospholipid metabolism	PLD1	Up	Cervical cancer	178
LncRNA SLNCR1	Phospholipid metabolism	PLA2	Up	Non-small cell lung cancer	181
LncRNA HULC	Cholesterol	FOXM1	Up	Hepatocellular carcinoma	185
LncRNA UCA1	Palmitic acid	ERK/MMP-9	Up	Gastric cancer	190-192
LncRNA ANRIL	Phospholipid	/	/	Large cell lung cancer	193

**Table 2:** Summary of ferroptosis-associated lncRNAs in cancer

LncRNA	Associated type of effect	Target	Influence to target	Cancer type	Reference
LncRNA UCA1	Ferroptosis	GSH	Up	Bladder cancer	198
LncRNA NEAT1	Ferroptosis	ACSL4	Down	Non-small cell lung cancer	204
LncRNA NEAT1	Ferroptosis	SLC7A11/GPX4	Up	Non-small cell lung cancer	204
LncRNA H19	Ferroptosis	Nrf2	Up	Ovarian cancer	206
LncRNA KRAL	Ferroptosis	Nrf2	Down	Hepatocellular carcinoma	207
LncRNA MALAT1	Ferroptosis	Nrf2	Down	Multiple myeloma	208
LncRNA PVT1	Ferroptosis	TFR1	Up	Hepatocellular carcinoma	212
LncRNA MT1DP	Ferroptosis	Nrf2	Down	Non-small cell lung cancer	214
LncRNA P53RRA	Ferroptosis	p53	Down	Lung cancer	216
LncRNA MEG3	Ferroptosis	p53	Up	Non-small cell lung cancer	218, 219
LINC00618	Ferroptosis	SLC7A11	Down	Acute myeloid leukemia	220
LINC00336	Ferroptosis	Cys	Up	Lung cancer	221
LncRNA GABPB1-AS1	Ferroptosis	ROS	Up	Hepatocellular carcinoma	224
LncRNA Gas5	Ferroptosis	ROS	Down	Melanoma	225

## Abbreviations

FAs: fatty acids
TG: triacylglycerol
PL: phospholipids
LPO: lipid peroxidation
ROS: reactive oxygen species
PUFA: polyunsaturated free fatty acids
lncRNAs: long noncoding RNAs
MVA: mevalonate
HMGCR: hydroxymethylglutaryl-coenzyme A reductase
SQLE: squalene epoxidase
LDLR: low-density lipoprotein receptor
GPX4: glutathione peroxidase 4
SREBP2: sterol regulatory element-binding protein 2
ER: endoplasmic reticulum
BC: breast cancer
BCSC: breast cancer stem cells
PCBP2: poly(rC) binding protein 2
NSCLC: non-small cell lung cancer
CE: cholesterol ester
ACAT: cholesterol acyltransferase
ABC: ATP binding cassette
LXRs: liver X receptors
RXRα: retinoid X receptors alpha
DR: direct repeat
GBM: glioblastoma
ABCA1: ATP-binding cassette transporter A1
MPT: mitochondrial permeability transition
EZH2: enhancer of zeste homolog 2
ACLY: ATP citrate lyase
ACC: acetyl-CoA carboxylase
FASN: fatty acid synthase
SCD: stearoyl-CoA desaturase
CoA: coenzyme A
GC: gastric cancer
NPC: nasopharyngeal carcinoma
SRPK2: serine/arginine protein-specific kinase 2
SR: serine/arginine
MMP-9: matrix metalloproteinase-9
ceRNA: competitive endogenous RNA
FAT/CD36: fatty acid translocase
FATP: fatty acid-binding protein
oxLDL: oxidized low-density lipoprotein
CPT1: carnitine palmityl transferase 1
FAO: fatty acid oxidation
TAM: tumor-associated macrophages
FFA: free fatty acid
HCC: hepatocellular carcinoma
EMT: epithelial-mesenchymal transition
AML: acute myeloid leukemia
TC: thyroid cancer
ACSL: acyl-CoA synthetase long-chain family members
MSCs: mesenchymal stem cells
LD: lipid droplets
LPA: lysophosphatidic acid
GPAT: glycerol 3-phosphate acyltransferase
FA-CoA: fatty acyl-CoA
PA: phosphatidic acid
AGPAT: acylglycerophosphate acyltransferase
PAP or Lipin: phosphatidic acid phosphohydrolase
DG: diacylglycerol
DGAT: diacylglycerol acyltransferase
ATGL: triglyceride lipase
HSL: hormone-sensitive lipase
MAGL: monoacylglycerol lipase
SphKs: sphingosine kinases
S1P: sphingosine-1-phosphate
PTC: papillary thyroid carcinoma
PLD: phospholipase D
PC: phosphatidylcholine
PLA2: phospholipase A2
DNMT1: DNA (Cytosin-5-)-methyltransferase 1
LOOH: lipid hydrogen peroxide
GSH: glutathione
GLS2: glutaminase2
NADPH: nicotinamide adenine dinucleotide phosphate
GR: glutathione reductase
GSSG: oxidized glutathione
Nrf2: Erythroid 2-related factor 2
TF: transferrin
TFR: transferrin receptor
LIP: labile iron pool
STEAP3: six-transmembrane epithelial antigen of the prostate 3
FTL: ferritin light chain
FPN1: ferroportin 1
LSH: lymphoid specific helicase
CBS: cystathionine β-synthase
TSP: transsulfuration pathway
G6PD: glucose 6-phosphate dehydrogenase
NOX4: NADPH oxidase 4
